# Delayed Dopamine Dysfunction and Motor Deficits in Female Parkinson Model Mice

**DOI:** 10.3390/ijms20246251

**Published:** 2019-12-11

**Authors:** Yuan-Hao Chen, Vicki Wang, Eagle Yi-Kung Huang, Yu-Ching Chou, Tung-Tai Kuo, Lars Olson, Barry J. Hoffer

**Affiliations:** 1 Department of Neurological Surgery, Tri-Service General Hospital, National Defense Medical Center, Taipei 11490, Taiwan; 2 Graduate Institute of Medical Sciences, National Defense Medical Center, Taipei 11490, Taiwan; y103110@gmail.com; 3 Department of Pharmacology, National Defense Medical Center, Taipei 11490, Taiwan; eyh58@mail.ndmctsgh.edu.tw; 4 School of Public Health, National Defense Medical Center, Taipei 11490, Taiwan; trishow@mail.ndmctsgh.edu.tw; 5 Graduate Institute of Computer and Communication Engineering, National Taipei University of Technology, Taipei 10608, Taiwan; k912225@yahoo.com.tw; 6 Department of Neuroscience, Karolinska Institute, 17177 Stockholm, Sweden; Lars.Olson@ki.se; 7 Department of Neurosurgery, Case Western Reserve University School of Medicine, Cleveland, OH 44106, USA

**Keywords:** Parkinson’s disease, MitoPark mouse, gender, estrogen

## Abstract

This study analyzed gender differences in the progressive dopamine (DA) deficiency phenotype in the MitoPark (MP) mouse model of Parkinson’s disease (PD) with progressive loss of DA release and reuptake in midbrain DA pathways. We found that the progressive loss of these DA presynaptic parameters begins significantly earlier in male than female MP mice. This was correlated with behavioral gender differences of both forced and spontaneous motor behavior. The degeneration of the nigrostriatal DA system in MP mice is earlier and more marked than that of the mesolimbic DA system, with male MP mice again being more strongly affected than female MP mice. After ovariectomy, DA presynaptic and behavioral changes in female mice become very similar to those of male animals. Our results suggest that estrogen, either directly or indirectly, is neuroprotective in the midbrain DA system. Our results are compatible with epidemiological data on incidence and symptom progression in PD, showing that men are more strongly affected than women at early ages.

## 1. Introduction

Parkinson’s disease (PD) is the second most common progressive neurodegenerative disease with a broad spectrum of motor and non-motor features resulting from progressive loss of dopaminergic (DAergic) neurons in the substantia nigra pars compacta (SNc) [1,2]. The etiologies of different types of PD are mostly genetic [3] and may include susceptibility genes interacting with factors including oxidative stress, mitochondrial dysfunction, protein mishandling, and epigenetics [4]. Mitochondrial dysfunction and energy failure are implicated as the cause of death of DA neurons in PD [5,6,7,8,9,10]. Toxins used to model PD, such as MPTP and rotenone, impair respiratory chain function by inhibiting complex I [11,12,13,14,15]. In further support for a “mitochondrial hypothesis” for PD pathophysiology, Bender et al. [16] reported higher levels of mitochondrial DNA deletions in nigral neurons from PD patients. Moreover, both Bender et al. [16] and Kraytsberg et al. [17] reported higher levels of mitochondrial DNA deletions in nigral neurons in aged humans with sharp elevations starting shortly before age 70. This correlates with age being a known major risk factor for PD. A better understanding of the progressive cellular events that precede the appearance of behavioral symptoms will be critical for the early diagnosis of PD and development of more effective treatment strategies.

It has been suggested that gender is an important factor in the development of PD. The disease is more common in men than in woman by an approximate ratio of 1.5–2:1 [18,19]. In addition to prevalence, several other PD-coupled parameters differ between men and women, including onset of symptoms, types of motor and non-motor symptoms, medication use, the effect size of PD risk factors, levodopa bioavailability, neuropsychiatric, and cognitive changes, development of hallucinations, caregiver utilization and reliance, and the quality of life [20,21]. In women, the age of PD onset has shown a positive correlation with fertility. Sex hormones, especially estrogen, may thus influence PD pathogenesis and be an important gender differentiation factor [22,23]. Here we use the MitoPark (MP) mouse model of PD where the mitochondrial transcription factor, TFAM, is specifically deleted in midbrain dopamine (DA) neurons. There is subsequent progressive degeneration of midbrain DA neurons which project to both striatum and extrastriatal telencephalic sites. Since the TFAM deletion is driven by the DAT promoter, neurons that do not express DAT are spared. Ovariectomy was used to address gender differences and the possible protective effects of estrogen on the time course of degeneration of DA neurons.

## 2. Results

**Figure 1:**
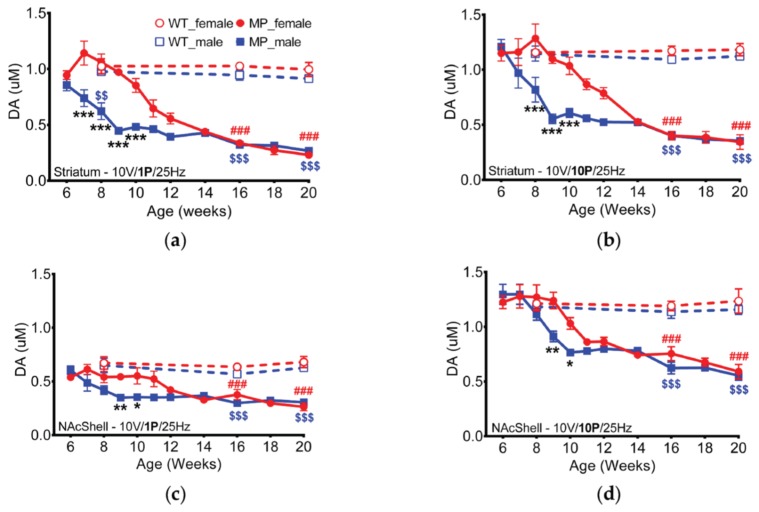
Dopamine concentration, measured in striatum with 10 V stimulation intensity, declined gradually with age in MitoPark mice. (**a**) In striatum with tonic (1 P) stimulation, the two sexes differ significantly at 7, 8, 9 and 10 weeks of age. (**b**) Phasic (10 P) stimulation-induced DA release. Male and female MP mice differ significantly at 8, 9 and 10 weeks of age. (**c**) In NAc shell tonic (1 P) and (**d**) phasic (10 P) stimulation showed that the two sexes of MP mice were significantly different at 9 and 10 weeks of age. Two-way analysis of variance (ANOVA) followed by a Bonferroni post hoc test for multiple comparisons; * *p* < 0.05, ** *p* < 0.01, *** *p* < 0.001 MitoPark_female vs. MitoPark_male; # *p* < 0.05, ### *p* < 0.001 WT_ female vs. MitoPark_female; $$ *p* < 0.01; $$$ *p* < 0.001 WT_male vs. MitoPark_male.

DA release rate was measured by fast scan cyclic voltammetry (FSCV) in ex vivo (in vitro) brain slices. The capacity of axon terminals to release DA was assessed by using one single pulse (for tonic) or 10 pulses (for phasic) stimulation delivered at 25 Hz under 10 volts stimulation intensities. There was no impairment in 6 weeks old MP mice compared to WT mice. In striatum, both tonic and phasic release were significantly different between male and female MP mice at eight, nine, and 10 weeks of age (Figure 1a,b, *p* < 0.001). Dopaminergic transmission in the shell of nucleus accumbens was also surveyed by ex vivo FSCV. We found that both tonic and phasic release differed significantly between male and female MP mice at nine and 10 weeks of age (Figure 1c,d, *p* < 0.01). The DA release in brain slices from male MP mice started to decline at seven weeks of age, while release in brain slices from female MP mice seemed to have a later decline at 10 weeks. In MP mice there were significant differences at 16 and 20 weeks compared with WT mice (Figure 1a,b, *p* < 0.001). There was no difference between male and female wild type (WT) mice at 12–20 weeks of age.

**Figure 2:**
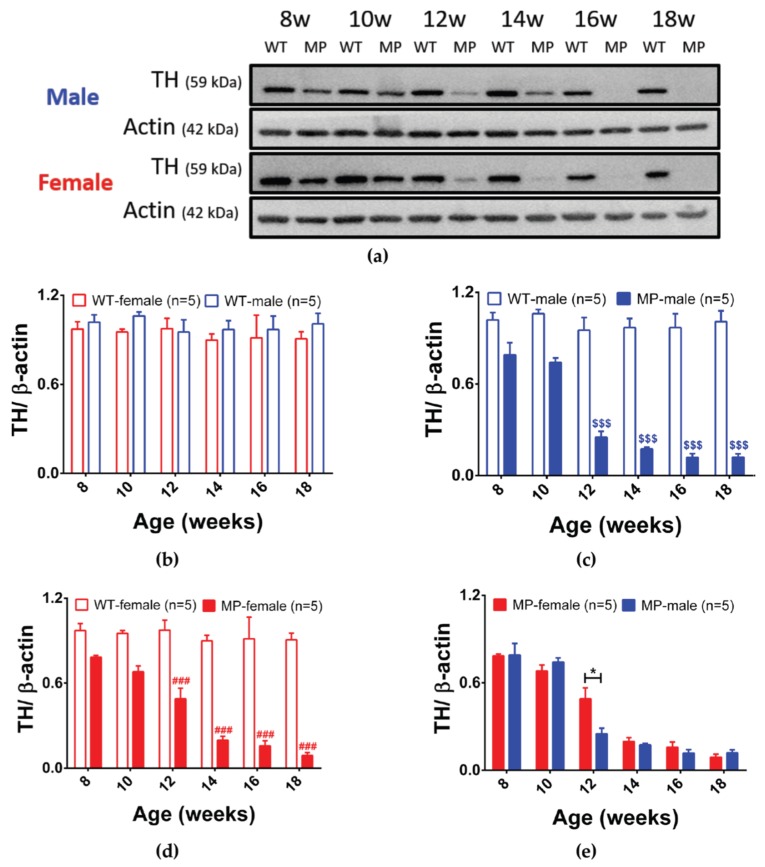
TH protein expression levels in female and male WT and MP mouse striatum. (**a**) TH protein expression in WT and MP mice. β-actin was used as a loading control. (**b**) Quantification of TH protein in male and female WT mice, did not reveal gender differences. Comparison of (**c**) male and (**d**) female TH expression between WT and MP mice. Both sexes are significantly different from controls at 10 weeks and marked decreases are seen at 12 weeks. (**e**) Male and female MP mice have different TH protein levels at 12 weeks. One-way analysis of variance (ANOVA) followed by a Bonferroni post hoc test for multiple comparisons; * *p* < 0.05 MitoPark_female vs. MitoPark_male; # *p* < 0.05, ### *p* < 0.001 WT_ female vs. MitoPark_female; $ *p* < 0.05; $$$ *p* < 0.001 WT_male vs. MitoPark_male.

The decline in DAergic neuron function was further investigated by measuring tyrosine hydroxylase (TH) protein in striatum (Figure 2a). In WT mice, there was no difference in TH expression levels between genders (Figure 2b). Compared with WT mice, the TH expression level in MP mice started to decrease significantly at 10 weeks (Figure 2c,d, both *p* < 0.05) and was markedly decreased at 12 weeks of age (Figure 2c,d, both *p* < 0.001) in both male and female MP mice. Interestingly, TH expression levels in female MP mice were significantly higher than in male MP mice at 12 weeks (Figure 2e, *p* < 0.05).

**Figure 3:**
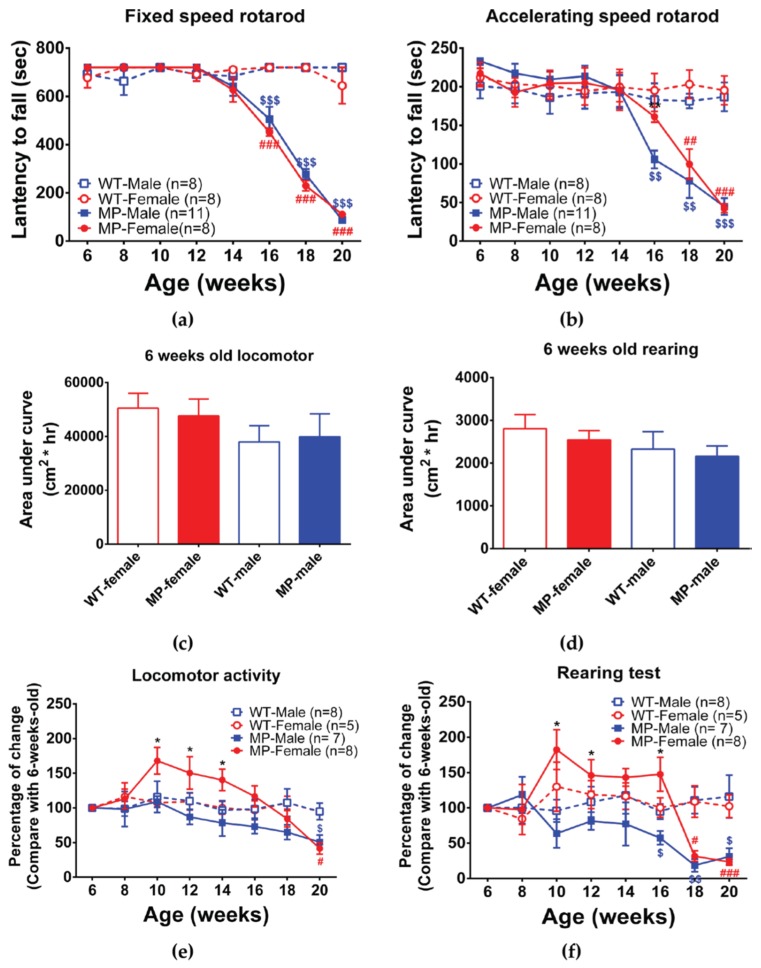
Non-spontaneous (motivated) and spontaneous motor behavior of male and female adult WT and MP mice. (**a**) Fixed speed rotarod, both male and female MP mice performed as well as WT mice up to 14 weeks of age. At the age of 16 weeks, MP mice showed a significant decline with no difference between the two sexes. (**b**) Accelerating speed rotarod. Male MP mice are significantly different from WT mice at 16 weeks, which is earlier than female MP mice. (**c**) Locomotor activity during 24 h in male and female WT and MP mice at the age of six weeks. There is no difference between groups. All mice were used. (**d**) Rearing during 24 h in male and female WT and MP mice at the age of 6 weeks. There is no difference between groups. All mice were used. (**e**) Percent change of locomotor activity, from 6 weeks to 20 weeks. Male and female MP mice differ significantly at 10 to 14 weeks. (**f**) Percent change of rearing from six weeks. Male and female MP mice differ at 10, 12 and 16 weeks. Two-way ANOVA [followed by a Bonferroni post hoc test for multiple comparisons; * *p* < 0.05, ** *p* < 0.01, *** *p* < 0.001 MP-Female vs. MP-Male; # *p* < 0.05, ## *p* < 0.01, ### *p* < 0.001 WT-Female vs. MP-Female; $ *p* < 0.05, $$ *p* < 0.01, $$$ *p* < 0.001 WT-male vs. MitoPark-male.

We monitored spontaneous activity, including 24 h locomotor activity and rearing, as well as non-spontaneous (motivated) activity using the fixed speed and accelerating speed rotarod to detect coordination and balance functions. MP mice were found to perform as well as WT mice up to 14 weeks of age on the fixed, and the accelerating speed rotarod tests. At 16 weeks, both male and female MP mice were impaired in the fixed speed rotarod, and the impairment was further increased from 16 to 18 weeks of age (Figure 3a). No difference was found between male and female MP mice. In contrast, on the accelerating speed rotarod male MP mice had a significantly reduced latency to fall compared to female MP mice at 16 weeks of age (Figure 3b, *p* < 0.01), but not at later ages. Interestingly, female MP mice did not differ from female WT mice at 16 weeks of age in the accelerating rotarod but started to decrease performance at the age of 18 weeks (Figure 3b).

The data on spontaneous activity were normalized using six weeks old data as a control because we had already determined that there is no significant difference between male and female WT or MP mice in locomotor activity and rearing at this age (Figure 3c,d). There were no significant differences in performance between male and female WT mice in any of the spontaneous measures during 6–20 weeks of age (Figure 3e,f). However, the data indicate that male and female MP mice have different behaviors from 10 weeks to 14 weeks of age (Figure 3e). Female MP mice had a higher locomotor activity than male MP mice before the age of 16 weeks, but both MP sexes had decreased locomotor activity at the ages of 18 and 20 weeks (Figure 3e,f). In the rearing test, there were changes similar to locomotor activity. Both male and female MP mice had significant differences at 10 (Figure 3f, *p* < 0.05), 12 (Figure 3f, *p* < 0.05) and 16 weeks (Figure 3f, *p* < 0.01) of age. After the age of 16 weeks, rearing activity by both MP sexes started to decline and there were no gender differences.

**Figure 4:**
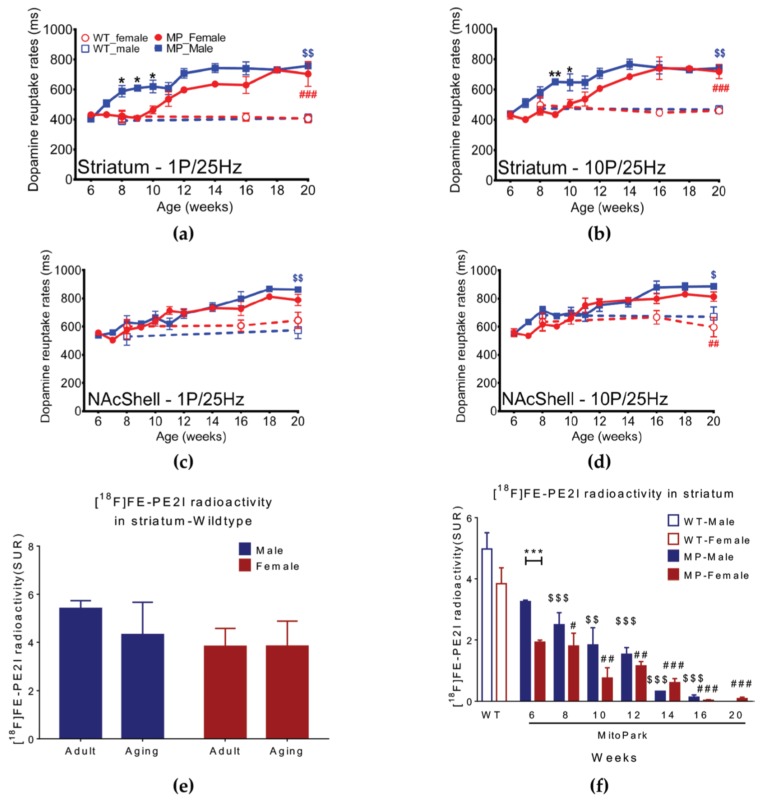
Sex differences of dopamine clearance rate in MP mice. DA reuptake efficacy (**a**) following tonic stimulation in striatum is less in male than female MP mice at eight, nine, and 10 weeks. At 11 weeks there is no gender difference. (**b**) With phasic stimulation, the male and female MP mice differ at 9 and 10 weeks. In NAc shell (**c**) tonic (1 P) and (**d**) phasic (10 P) stimulation does not reveal any gender differences. (**e**) PET imaging using the DA transporter ligand [^18^F]FE-PEI in striatum does not reveal gender differences in WT mice. (**f**) PET shows that the binding of the DA transporter ligand in striatum differs significantly between genders in six-week old MP mice and a similar trend is seen at later ages. The value of the WT group is the average from adult and aging mice. Two-way analysis of variance (ANOVA) followed by a Bonferroni post hoc test for multiple comparisons; * *p* < 0.05, ** *p* < 0.01, *** *p* < 0.001 MitoPark_Female vs. MitoPark_Male; # *p* < 0.05, ## *p* < 0.01, ### *p* < 0.001 WT vs. MitoPark_Female; $ *p* < 0.05, $$ *p* < 0.01, $$$ *p* < 0.001 WT vs. MitoPark_Male.

There were significant differences in DA reuptake in striatum between male and female MP mice at 8 (Figure 4a, *p* < 0.05) and 10 (Figure 4a, *p* < 0.05) weeks of age following tonic stimulation. Phasic stimulation also revealed MP gender differences in striatum at nine and 10 weeks of age (Figure 4b, *p* < 0.05). Both tonic and phasic reuptake differed significantly at 16 and 20 weeks between female WT and MP mice but not between male and female MP mice. In nucleus accumbens (NAc) shell, neither tonic (1 P) nor phasic (10 P) stimulation differed between genders in MP mice (Figure 4c,d).

We next studied positron emission tomography (PET) imaging using the DA transporter ligand [^18^F]FE-PEI in striatum. There were no differences between male and female WT mice (Figure 4e). However, the DA reuptake efficacy in striatum differed between male and female MP mice at 6 weeks of age (Figure 4f, *p* < 0.001). After the age of eight weeks, DA reuptake efficacy in striatum in both MP sexes start to decline with no significant gender difference (Figure 4f).

**Figure 5:**
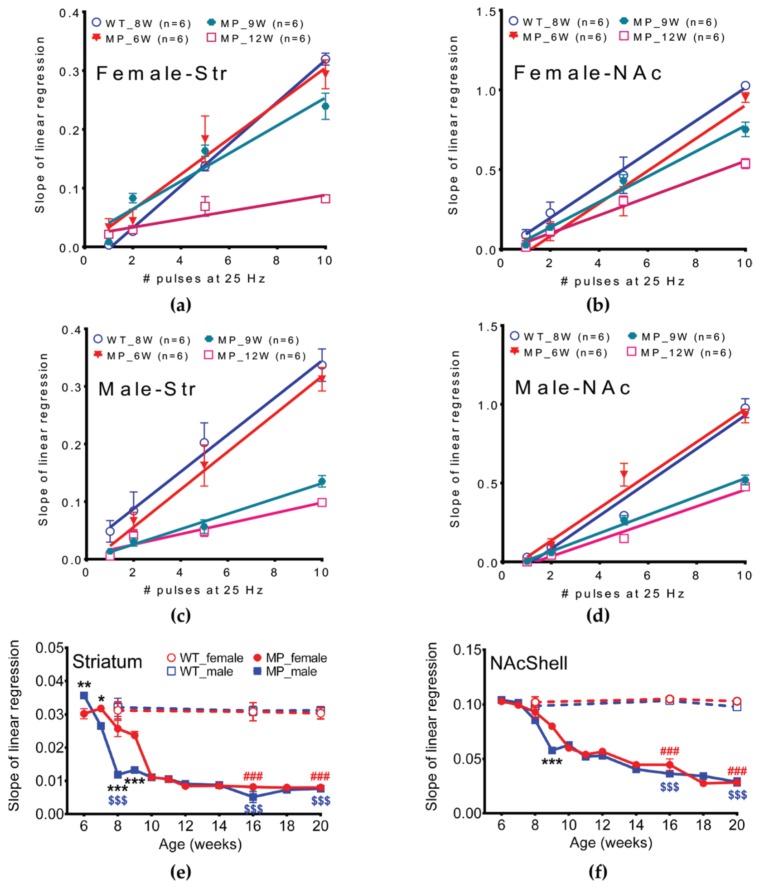
Release probability of dopamine in male and female MP mice. A decline of release probability with age of MitoPark mice is found in both (**a**) striatum of female MP, (**b**) NAc shell of female MP, (**c**) striatum of male MP and (**d**) NAc shell of male MP. (**e**) In striatum, the value of the slope decreased by eight weeks of age in males while significant changes in slope could not be found before nine weeks of age in females. (**f**) DA release probabilities in NAc shell showed that male and female MP mice differed significantly at the age of nine weeks. Two-way analysis of variance (ANOVA) followed by a Bonferroni post hoc test for multiple comparisons; * *p* < 0.05, ** *p* < 0.01, *** *p* < 0.001 MitoPark_Female vs. MitoPark_Male; ### *p* < 0.001 WT vs. MitoPark_Female; $$$ *p* < 0.001 WT vs. MitoPark_Male.

Dopamine release probabilities can be expressed as the slope of the linear regression line (Figure 5a–d). Both striatal and NAc shell slopes decreased as MP mice of both genders aged (Figure 5a–d). In striatum, the value of the slope decreased by eight weeks of age in males while significant changes in slope could not be found before nine weeks of age in females (Figure 5e, *p* < 0.001). DA release probabilities in NAc shell showed that male and female MP mice differed significantly at the age of nine weeks (Figure 5f, *p* < 0.001).

Based on the above data, we thus found that there are gender differences of DA release, DA reuptake rate, TH protein expression in striatum, and motor activity in MP mice. To identify part of the reasons for these differences, ovariectomy was performed in five-week old female MP mice to determine whether the differences were related to female sex hormones.

**Figure 6:**
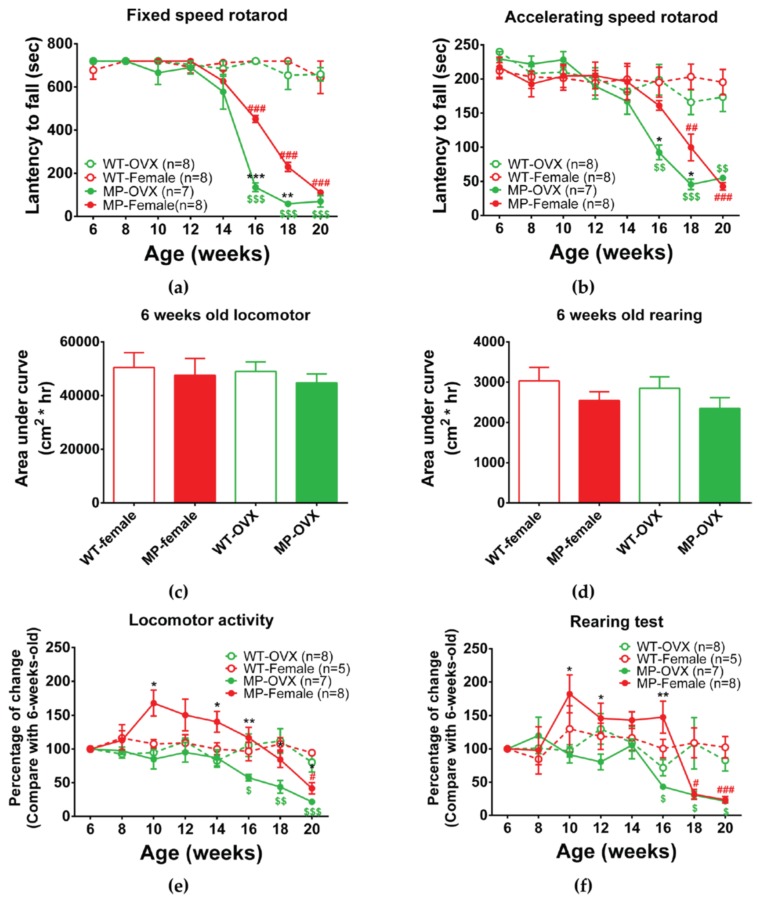
The influence of ovariectomy on non-spontaneous (motivated) and spontaneous motor behavior of female WT or MP mice. (**a**) Fixed speed rotarod test shows that OVX MP mice do not differ from OVX WT mice before the age of 14 weeks. The activity of OVX MP starts to decline from 16 weeks of age and is significantly different from that of female MP mice at 16 and 18 weeks. (**b**) Similar results from the accelerating speed rotarod test. OVX MP mice start to decline at the age of 16 weeks compared to OVX WT mice and are significantly different from female MP mice at 16 and 18 weeks. (**c**) Total locomotor activity during 24 h of OVX, female WT, and MP mice at the age of 6 weeks. There was no difference between the groups. (**d**) Rearing events during 24 h of OVX, female WT, and MP mice at the age of six weeks. There is no difference between the groups. All the mice were used for the following test. (**e**) Percentage change in locomotor activity, compared to activity at six weeks. OVX and female MP mice are significantly different at 10 weeks and 14 to 20 weeks. (**f**) Percent change of rearing since week six. At 10, 12 and 16 weeks rearing by ovariectomized MP mice is significantly less common than in non-ovariectomized female MP mice. Two-way ANOVA [followed by a Bonferroni post hoc test for multiple comparisons; * *p* < 0.05, ** *p* < 0.01, *** *p* < 0.001 MP-Female vs. MP-OVX; # *p* < 0.05, ## *p* < 0.01, ### *p* < 0.001 WT-Female vs. MP-Female; $ *p* < 0.05, $$ *p* < 0.01, $$$ *p* < 0.001 WT-OVX vs. MitoPark-OVX.

We found that ovariectomized female (OVX) MP mice performed as well as WT mice and female MP mice up to 14 weeks of age on the fixed, and accelerating speed rotarod. At the age of 16 weeks, OVX MP females started to decrease activity in both tests (Figure 6a,b). Interestingly, the OVX female MP and female MP mice differed significantly with regard to both fixed speed and accelerating rotarod at 16 (Figure 6a, *p* < 0.001; Figure 6b, *p* < 0.05) and 18 weeks of age (Figure 6a, *p* < 0.01; Figure 6b, *p* < 0.05). However, at the age of 20 weeks, OVX female MP mice no longer differed from female MP mice in terms of rotarod performance.

Locomotor activity and rearing during 24 h did not differ between OVX and non-operated female MP mice at the age of six weeks (Figure 6c,d). There were no significant changes or differences between female and OVX female WT mice in any of the spontaneous activities at 6–20 weeks of age (Figure 6e,f). Importantly, locomotor activity was significantly different at the ages of 10 (Figure 6e, *p* < 0.001), 12 (Figure 6e, *p* < 0.001) and 14 weeks (Figure 6e, *p* < 0.05) between OVX and non-operated female MP mice. In addition, rearing revealed differences at 10 (Figure 6f, *p* < 0.005), 12 (Figure 6f, *p* < 0.001), and 16 weeks (Figure 6f, *p* < 0.01) of age between OVX and non-operated female MP mice.

## 3. Discussion

The MitoPark model [24] allows for studies of the role of DA degeneration per se, studies of DA neuropathology, and behavioral effects over an extended time course [25,26,27]. The progressive loss of the MitoPark midbrain DA system elicits a useful phenocopy of human PD, in terms of behavior, neurochemistry, and histology.

The interpretation of results from more traditional experiments with neurotoxins are complicated by additional pharmacological effects in DA neurons, effects on non-DA cell types, technical variability of toxin administration, etc. However, it has been demonstrated that the stimulation of estrogen receptors is neuroprotective in the MPTP model of PD in mice [28].

The “reproducibility” of the MP phenotype is another specific advantage of the genetic model. The data here are strikingly similar to findings reported almost a decade ago on motor behavior and DA systems, albeit there was no differentiation in these earlier studies as to MP gender. Interestingly, the difference between the nigrostriatal and ventral tegmental nucleus (VTA) mesolimbic system in terms of DA presynaptic functions, reported here parallel changes in DA levels and TH immunocytochemistry seen earlier [24].

We have previously shown that the synthesis, release, and reuptake of DA are all reduced in MP mice, prior to loss of the neurons themselves. Here, we show that progressive loss of these phenotypic properties in MP mice is found earlier in males than females. We also show that the loss of release, reuptake and TH are all correlated with loss of motivated and spontaneous behaviors that occurs earlier in males than females. We hypothesize that the sex difference in MP mice relate to female sex hormones. This hypothesis is supported by our findings that after ovariectomy, female MP mice show progressive changes in DA presynaptic dynamics, such as release and reuptake as well as behavioral impairment with time courses similar to those of male MP animals, in contrast to the delayed changes seen in intact MP females. However, it should be noted that ovariectomy produces a number of changes in both brain and periphery that may underlie some of these changes.

It has been reported that estrogen, more precisely, 17β-estradiol (E2) has neuroprotective effects on nigrostriatal DA neurons and can modulate monoamine oxidase [29]. Estrogen might also partially inhibit interleukin 6 (IL-6) production, which increases neuroinflammatory responses, and microglial activation, which play key roles in PD development [30,31]. This is supported by studies showing that neuroinflammation and microglial activation play a role in the progression of PD [32,33,34,35].

There is a great deal of evidence that Parkinson’s disease (PD) affects men significantly more than women. As noted above, the ratio varies among studies and may also vary by country, but on average most reports indicate that about 1.5 times more men get PD than do women [36], although even greater gender disparities have been reported. In addition to the difference in incidence, there are differences in the characteristics of the disease. For example, men tend to show more dyskinesia and depression, whereas woman exhibit more rigidity and rapid eye movement abnormalities [37].

Several possible explanations have been offered for the difference in incidence, including genetic factors, differences in lifestyle (e.g., exposure to herbicides or head trauma), and different reactions to stressful events (for review, see [38]). However, it is most commonly assumed that the male:female difference in the incidence of PD is related to the neuroprotective effects of estrogens in females. This is consistent with the observation that men exhibit PD at an earlier age and that the difference in incidence wanes and ultimately disappears after menopause [36]. This finding is also observed in animal models [39], and the neuroprotective effects of estrogens on dopamine (DA) neurons that have been observed both in animal models [40] and in vitro [41]. This may also relate to the observed inhibitory effect of estrogens on the high affinity DA transporter [42], which in turn would reduce the oxidative stress that DA can cause via its conversion to free radicals.

Estrogen receptor β activation can affect transcription of mitochondrial genes via interaction with estrogen response elements (ERE) or protein-protein interactions with mitochondria imported transcription factors [43]. Mitochondria are critically involved in cellular physiology including calcium homeostasis, metabolism, apoptosis and ATP production by the electron transport chain (ETC) [44]. At respiratory complexes I and III of the ETC, mitochondria produce reactive oxygen species (ROS) [45]. Therefore, close to the menopause or at the pre-menopause state, estrogen decline may affect the functional state of mitochondria, causing declines of cellular functions and aging [46]. Our findings suggest that the initial effects of lack of trancription factor TFAM in MitoPark DA neurons can be partially counteracted by estrogen. Interestingly, we found that female MP mice initially were more active than WT mice (Figure 3e/f and Figure 6e/f). This may be due to leakage of DA from the first degenerating striatal DA terminals. That this is seen in females but not in males adds to the view that estrogen protects the TFAM-lacking DA neurons, presumably by prolonging the time course for degeneration.

It appears from our data that male MP mice have the slowest DA reuptake rate, while PET imaging shows that female MP mice bind less amounts of the DAT ligand than male MP mice (at least between six and 12 weeks). Nevertheless striatal DA reuptake does not differ between males and females at the age when the PET studies were done. DAT imaging with FE-PE2I PET allows excellent basic diagnostic differentiation in early-stages of Parkinson’s disease [47]. Although beyond the scope of the present study, the apparent gender difference in DAT binding of the used radioactive ligand in our mouse PD model therefore deserves further studies.

Unlike the case for most forms of PD, the pathology in the MP mouse brain is presumably restricted to systems expressing DAT. It can therefore be concluded that the gender difference detected in the MP mice and the lack of gender difference following ovariectomy are both due to effects of estrogen directly on DAT-expressing neurons.

## 4. Materials and Methods

A number of the methods detailed below have been described in our recent publication [48]. They are summarized here, however, to facilitate reproducibility of our data by other laboratories, specifically for MitoPark breeding and generation, as well as for behavior and FSCV techniques.

### 4.1. Animals

The breeding scheme for generating MitoPark mice has been described previously [24,25,26]. Briefly, animals on a C57BL6 background, in which the DA transporter (DAT) promoter was used to drive cre-recombinase expression, were crossed with mice in which the Tfam gene had been loxP-flanked. Breeding pairs to generate MP mice were sent to the National Defense Medical Center (NDMC) in Taiwan from a colony maintained at the NIDA (NIH) Intramural Program. Male and female MP mice used in these experiments were heterozygous for DAT-cre expression (DAT/DAT^cre^) and homozygous for the loxP-flanked Tfam gene (Tfam^loxP^/Tfam^loxP^). Age-matched wild type mice were used as controls.

A total of 185 MP mice (87 males, 84 females, 14 females + OVX) and 105 control (WT) mice (46 males, 43 females, 16 females + OVX) were used (Table 1). The mice were housed at 25 °C with a 12/12 light/dark cycle and continuous water and food supply. All efforts were made to reduce animal suffering and to minimize the number of animals used. The procedures of this study were approved by the NDMC Animal Care and Use Committee and followed the NDMC Guidelines for the Care and Use of Laboratory Animals (IACUC 18-275 (27 08 2018) and IACUC 19-198 (29 05 2019)).

### 4.2. NAc and Striatal Brain Slice Preparation

Brain slices were prepared as described previously [49,50]. After decapitation, the brain was removed and immersed in oxygenated (95% O_2_/5% CO_2_) cold cutting solution. The tissues were cut into coronal slices (300 μm) using a tissue slicer (VT 100, Leica, Wetzlar, Germany). The slices were then transferred to a holding chamber filled with oxygenated artificial CSF solution atc30 °C at least for 30 min.

### 4.3. Fast Scan Cyclic Voltammetry and Dopamine Measurements in Brain Slices

FSCV ex vivo recordings were performed as described previously [51,52]. Slices of brain tissue were kept in a chamber perfused with artificial CSF. Carbon fibers were lowered into striatum or NAc and positioned between the separated tips of a bipolar stimulating electrode. DA signals used in the statistical analyses matched the expected voltammetric profile for DA [53]. A single pulse DA release evoked by a single pulse (tonic release) represent the underlying release per pulse and DA re-uptake by DAT [54]. DA signals were converted to DA concentrations using calibration protocol for each electrode. We used a single pulse (for tonic) or 10 pulses (for phasic) stimulation at 25 Hz and stimulation intensities of 1–10 volts.

DA uptake was assessed using a published protocol (WinWCP; Dr. John Dempster, Strathclyde Institute for Biomedical Sciences, Glasgow, UK; http://spider.science.strath.ac.uk), and an index of DA clearance efficiency (V_max_/K_m_) DA transporter (DAT) was also obtained [49,55].

DA Release probability was calculated as previously demonstrated [48]. Data were fit to a linear regression model (Prism 6.02; GraphPad, San Diego, CA, USA), where the slope represents a change in DA concentration per pulse [49,51].

### 4.4. Rotarod Test

Rotarod tests were performed to evaluate motor coordination and balance. In a training phase, mice were introduced to walking on the rotating rod one day before being tested. The training was completed when the mice were able to walk forward for 720 s at 15 rpm. Both fixed speed (20 rpm, cut-off time 720 s) and accelerating speed (from 5 rpm to 80 rpm within 240 s) rotarod tests were performed 3 times a day every 2 weeks. The time until the animal fell off the rotating rod was recorded by blinded observers.

### 4.5. Locomotor Activity and Rearing Test

Locomotor activity was evaluated using activity boxes (45 × 45 cm) in a low-noise experimental environment. DietGel^®^, a nutritional dietary supplement combining hydration and nutrition in a single serving was put into the boxes. Backward and forward movements were monitored with a grid of infrared beams over a 24-h period. The horizontal movements during 24 h were taken as distance traveled. The rearing test was performed to evaluate vertical movements and axial set. The number of rearings was recorded at the same time as the locomotor activity.

### 4.6. Western Blot

Mice were sacrificed by decapitation, and brains immediately cooled by immersion in liquid nitrogen for 6 s. The striatal tissue was homogenized in RIPA buffer (TAAR-ZBZ5, Biotools Co., Ltd., Taiwan) with a protease inhibitor cocktail (ab20111, Abcam, Cambridgeshire, United Kingdom). Protein concentration was measured using BCA protein assay kit II (K813, Biovision, Lausen, Switzerland). Subsequently, 30 µg protein in tissue lysates were diluted in 2X SDS buffer and denatured at 95 °C for 5 min. Proteins were then electrophoresed on a 12% SDS-polyacrylamide gel and electrotransferred onto a polyvinyl difluoride membrane. After 45 min incubation with blocking buffer (2% bovine serum albumin with tris-buffered saline with Tween 20 (TBST)), the membranes were incubated with primary antibodies TH (1:1000, rabbit, ab75875, Abcam, Cambridge, United Kingdom), beta-actin (1:5000, rabbit, ab8227, Abcam, Cambridge, United Kingdom.) overnight. After washing 3 times with TBST, the membranes were incubated with goat HRP-linked anti-rabbit IgG antibody (1:20,000, ab6721, Abcam, Cambridge, United Kingdom) for 1 h and then developed with an enhanced chemiluminescence (ECL) detection kit (Amersham Life Sciences, Piscataway, NJ, USA) and imaged with UVP ChemStudio Plus (Analytik Jena AG, Germany). All results were normalized to the levels of beta-actin used as the loading control, and the amount of immunoreactivity was calculated relative to the expression with the corresponding controls.

### 4.7. [^18^F]FE-PE2I PET Scan Imaging for Dopamine Transporter Function

Using an automatic synthesizer system (GE TRACERlab FX2 N), radioactive ^18^F nuclei were produced by a cyclotron. After separation, the tosylethyl-PE2I precursor was dissolved in anhydrous dimethyl sulfoxide, and reacted with k[^18^F]/ Kryptofix2.2.2 at −140 °C for five minutes, followed by purification using high performance liquid chromatography with a C18 column to obtain the PET ligand [^18^F]FE-PE2I.

MitoPark and WT mice were subjected to PET scans. Tail vein injections of 0.3 mCi [^18^F]FE-PE2I were delivered during anesthesia (5% isoflurane/oxygen mixture for induction, 2% for maintenance). Each injected mouse was first kept in a radiation prevention box for 20 min and then transferred to a PET scanner for static image scans with the energy window being set from 250 to 700 KeV. The 3D images of the brain were thus captured about 20 min after ligand injection.

Images were smoothened using a Gaussian algorithm. To minimize inconsistencies in the volume of interest (VOI) placement among the animals, magnetic resonance imaging (MRI) of the same age mouse brains were obtained and manually aligned with six re-constructed [^18^F]-PE2I PET images of mice to draw the VOI. The VOI was defined on reconstructed and summated PET images based on the MRI and a mouse brain atlas to determine the anatomical boundaries of striatum. The specific uptake ratio (SUR) was calculated as:

(1) $$\mathrm{Specific}\;\mathrm{uptake}\;\mathrm{ratio}\;(\mathrm{SUR})=\frac{\mathrm{VOI}\;\mathrm{standardized}\;\mathrm{uptake}\;\mathrm{value}\;\left[\mathrm{SUV}\right]\;\mathrm{value}-\mathrm{Background}\;\mathrm{SUV}\;\mathrm{value}}{\mathrm{Background}\;\mathrm{SUV}\;\mathrm{value}}$$

### 4.8. Statistics

Statistical analyses of data for DA release, DA reuptake, behavioral tests and western blot were performed using a two-way analysis of variance (ANOVA) followed by a Bonferroni post hoc test for multiple comparisons. All statistical tests were two-tailed and were performed using appropriate software (GraphPad Prism 6.02, GraphPad Scientific, San Diego, CA, USA). No animals were excluded, and no inclusion or exclusion criteria were determined. Tests for normality were not performed. A *p*-value < 0.05 using a two-tailed test was considered significant.

## 5. Conclusions

We conclude that MP mice that differ in no other way then gender have different PD development histories, with males having earlier behavioral symptoms than females, and that these differences parallel earlier impairment of presynaptic DA functions in male MP mice. Removal of the ovaries in female MP mice alters their DA disturbances and behavior impairments such that they begin earlier, with a similar time course as that of male MP mice. These observations are in agreement with other studies suggesting neuroprotective effects of estrogen and are also compatible with the clinical findings of a larger incidence of PD and an earlier symptom onset in males, compared to females. 

## Figures and Tables

**Table 1:** Numbers, gender, and age (weeks) of control and MitoPark mice used.

Age (Week)		6	7	8	9	10	11	12	14	16	18	20	Total Animals
**Group**		**FSCV (Figure 1, Figure 4a–d, and Figure 5)**											
WT	Male			3						3		3	9
	Female			3						3		3	9
	Female + OVX												0
MP	Male	3	3	3	3	5	3	3	3	3	3	3	35
	Female	3	3	3	3	5	3	3	3	3	3	3	35
	Female + OVX												0
		**WB (Figure 2)**											
WT	Male		3	3	3	3	3	3					18
	Female		3	3	3	3	3	3					18
	Female + OVX												0
MP	Male		3	3	3	3	3	3					18
	Female		3	3	3	3	3	3					18
	Female + OVX												0
		**Non-spontaneous activity (Figure 3 and Figure 6)**											
WT	Male	8											8
	Female	8											8
	Female + OVX	8											8
MP	Male	11											11
	Female	8											8
	Female + OVX	7											7
		**Spontaneous activity (Figure 3 and Figure 6)**											
WT	Male	8											8
	Female	5											5
	Female + OVX	8											8
MP	Male	8											8
	Female	8											8
	Female + OVX	7											7
		**PET (Figure 4e,f)**											
WT	Male	3 (adult, 6–11 W)						2 (aging, > 12 W)					3
	Female	2 (adult, 6–11 W)						2 (aging, > 12 W)					3
	Female + OVX												0
MP	Male	5	8	2	7	1	3		2				15
	Female	4	6	2	3	5	4		3				15
	Female + OVX												0

Some of the mice used in PET are reused at each time point. The numbers in the table are the values at each time point.

## Abbreviations

ANOVA	Analysis of variance
DA	Dopamine
DAergic	Dopaminergic
DAT	Dopamine transporter
DNA	Deoxyribonucleic acid
E2	17 beta-estradiol
FSCV	Fast scan cyclic voltammetry
HRP	Horseradish peroxidase
IL-6	Interleukin 6
I/O	Input/output
MP	MitoPark
MPTP	1-meth 1-4-phenyl-1236 tetrahydropyridine
MRI	Magnetic resonance imaging
NAc	Nucleus accumbens
OVX	Ovariectomy
PD	Parkinson’s Disease
PET	Positron emission tomography
SNc	Substantia nigra
SUR	Specific uptake ratio
SUV	standardized uptake value
TBST	Tris buffered saline with Tween20
TFAM	Mitochondrial transcription factor A
TH	Tyrosine hydroxylase
VOI	Volume of interest
VTA	Ventral tegmental nucleus
WT	Wild type

## References

[1] Hornykiewicz O. (2001). Chemical neuroanatomy of the basal ganglia--normal and in parkinson’s disease. J. Chem. Neuroanat..

[2] Thenganatt M.A., Jankovic J. (2014). Parkinson disease subtypes. JAMA Neurol..

[3] De Lau L.M.L., Breteler M.M.B. (2006). Epidemiology of parkinson’s disease. Lancet Neurol..

[4] Greenamyre J.T., Hastings T.G.J.S. (2004). Parkinson’s--divergent causes, convergent mechanisms. Science.

[5] Dauer W., Przedborski S. (2003). Parkinson’s disease: Mechanisms and models. Neuron.

[6] Dawson T.M., Dawson V.L. (2003). Molecular pathways of neurodegeneration in parkinson’s disease. Science.

[7] Ellis C.E., Murphy E.J., Mitchell D.C., Golovko M.Y., Scaglia F., Barceló-Coblijn G.C., Nussbaum R.L. (2005). Mitochondrial lipid abnormality and electron transport chain impairment in mice lacking α-synuclein. Mol. Cell. Biol..

[8] Mizuno Y., Ohta S., Tanaka M., Takamiya S., Suzuki K., Sato T., Oya H., Ozawa T., Kagawa Y. (1989). Deficiencies in complex i subunits of the respiratory chain in parkinson’s disease. Biochem. Biophys. Res. Commun..

[9] Shen J., Cookson M.R. (2004). Mitochondria and dopamine: New insights into recessive parkinsonism. Neuron.

[10] Ved R., Saha S., Westlund B., Perier C., Burnam L., Sluder A., Hoener M., Rodrigues C.M.P., Alfonso A., Steer C. (2005). Similar patterns of mitochondrial vulnerability and rescue induced by genetic modification of α-synuclein, parkin, and dj-1 in caenorhabditis elegans. J. Biol. Chem..

[11] Betarbet R., Sherer T.B., MacKenzie G., Garcia-Osuna M., Panov A.V., Greenamyre J.T. (2000). Chronic systemic pesticide exposure reproduces features of parkinson’s disease. Nat. Neurosci..

[12] Langston J.W., Ballard P., Tetrud J.W., Irwin I. (1983). Chronic parkinsonism in humans due to a product of meperidine-analog synthesis. Science.

[13] Mizuno Y., Sone N., Saitoh T. (1987). Effects of 1-methyl-4-phenyl-1, 2, 3, 6-tetrahydropyridine and 1-methyl-4-phenylpyridinium ion on activities of the enzymes in the electron transport system in mouse brain. J. Neurochem..

[14] Sherer T.B., Betarbet R., Testa C.M., Seo B.B., Richardson J.R., Kim J.H., Miller G.W., Yagi T., Matsuno-Yagi A., Greenamyre J.T. (2003). Mechanism of toxicity in rotenone models of parkinson’s disease. J. Neurosci..

[15] Smeyne R.J., Jackson-Lewis V. (2005). The MPTP model of parkinson’s disease. Mol. Brain Res..

[16] Bender A., Krishnan K.J., Morris C.M., Taylor G.A., Reeve A.K., Perry R.H., Jaros E., Hersheson J.S., Betts J., Klopstock T. (2006). High levels of mitochondrial DNA deletions in substantia nigra neurons in aging and parkinson disease. Nat. Genet..

[17] Kraytsberg Y., Kudryavtseva E., McKee A.C., Geula C., Kowall N.W., Khrapko K. (2006). Mitochondrial DNA deletions are abundant and cause functional impairment in aged human substantia nigra neurons. Nat. Genet..

[18] Baldereschi M., Di Carlo A., Rocca W.A., Vanni P., Maggi S., Perissinotto E., Grigoletto F., Amaducci L., Inzitari D. (2000). Parkinson’s disease and parkinsonism in a longitudinal study: Two-fold higher incidence in men. Neurology.

[19] Solla P., Cannas A., Marrosu M.G., Marrosu F. (2012). Dopaminergic-induced paraphilias associated with impulse control and related disorders in patients with parkinson disease. J. Neurol..

[20] Cabezas R., Ávila M., Gonzalez J., El-Bachá R.S., Báez E., García-Segura L.M., Jurado Coronel J.C., Capani F., Cardona-Gomez G.P., Barreto G.E. (2014). Astrocytic modulation of blood brain barrier: Perspectives on parkinson’s disease. Front. Cell. Neurosci..

[21] Colombo D., Abbruzzese G., Antonini A., Barone P., Bellia G., Franconi F., Simoni L., Attar M., Zagni E., Haggiag S. (2015). The “gender factor” in wearing-off among patients with parkinson’s disease: A post hoc analysis of deep study. Sci. World J..

[22] Gillies G.E., McArthur S. (2010). Independent influences of sex steroids of systemic and central origin in a rat model of parkinson’s disease: A contribution to sex-specific neuroprotection by estrogens. Horm. Behav..

[23] Gillies G.E., McArthur S. (2010). Estrogen actions in the brain and the basis for differential action in men and women: A case for sex-specific medicines. Pharmacol. Rev..

[24] Ekstrand M.I., Terzioglu M., Galter D., Zhu S., Hofstetter C., Lindqvist E., Thams S., Bergstrand A., Hansson F.S., Trifunovic A. (2007). Progressive parkinsonism in mice with respiratory-chain-deficient dopamine neurons. Proc. Natl. Acad. Sci. USA.

[25] Galter D., Pernold K., Yoshitake T., Lindqvist E., Hoffer B., Kehr J., Larsson N.G., Olson L. (2010). Mitopark mice mirror the slow progression of key symptoms and l-dopa response in parkinson’s disease. Genes Brain Behav..

[26] Good C.H., Hoffman A.F., Hoffer B.J., Chefer V.I., Shippenberg T.S., Bäckman C.M., Larsson N.-G., Olson L., Gellhaar S., Galter D. (2011). Impaired nigrostriatal function precedes behavioral deficits in a genetic mitochondrial model of parkinson’s disease. FASEB J..

[27] Sterky F.H., Lee S., Wibom R., Olson L., Larsson N.-G. (2011). Impaired mitochondrial transport and parkin-independent degeneration of respiratory chain-deficient dopamine neurons in vivo. Proc. Natl. Acad. Sci. USA.

[28] Nakaso K., Horikoshi Y., Takahashi T., Hanaki T., Nakasone M., Kitagawa Y., Koike T., Matsura T. (2016). Estrogen receptor-mediated effect of δ-tocotrienol prevents neurotoxicity and motor deficit in the MPTP mouse model of parkinson’s disease. Neurosci. Lett..

[29] Kelada S.N., Costa-Mallen P., Costa L.G., Smith-Weller T., Franklin G.M., Swanson P.D., Longstreth Jr W.T., Checkoway H. (2002). Gender difference in the interaction of smoking and monoamine oxidase b intron 13 genotype in parkinson’s disease. Neurotoxicology.

[30] Ray P., Ghosh S.K., Zhang D.-H., Ray A. (1997). Repression of interleukin-6 gene expression by 17β-estradiol: Inhibition of the DNA-binding activity of the transcription factors nf-il6 and nf-κb by the estrogen receptor. FEBS Lett..

[31] Vegeto E., Bonincontro C., Pollio G., Sala A., Viappiani S., Nardi F., Brusadelli A., Viviani B., Ciana P., Maggi A. (2001). Estrogen prevents the lipopolysaccharide-induced inflammatory response in microglia. J. Neurosci..

[32] Gerhard A., Pavese N., Hotton G., Turkheimer F., Es M., Hammers A., Eggert K., Oertel W., Banati R.B., Brooks D.J. (2006). In vivo imaging of microglial activation with [11c](r)-pk11195 pet in idiopathic parkinson’s disease. Neurobiol. Dis..

[33] Rodriguez-Pallares J., Parga J.A., Munoz A., Rey P., Guerra M.J., Labandeira-Garcia J.L. (2007). Mechanism of 6-hydroxydopamine neurotoxicity: The role of NADPH oxidase and microglial activation in 6-hydroxydopamine-induced degeneration of dopaminergic neurons. J. Neurochem..

[34] Suzuki K., Miyamoto T., Miyamoto M., Kaji Y., Takekawa H., Hirata K. (2007). Circadian variation of core body temperature in parkinson disease patients with depression: A potential biological marker for depression in parkinson disease. Neuropsychobiology.

[35] Tripanichkul W., Sripanichkulchai K., Finkelstein D.I. (2006). Estrogen down-regulates glial activation in male mice following 1-methyl-4-phenyl-1, 2, 3, 6-tetrahydropyridine intoxication. Brain Res..

[36] Georgiev D., Hamberg K., Hariz M., Forsgren L., Hariz G.M. (2017). Gender differences in parkinson’s disease: A clinical perspective. Acta Neurol. Scand..

[37] Miller I.N., Cronin-Golomb A. (2010). Gender differences in parkinson’s disease: Clinical characteristics and cognition. Mov. Disord..

[38] Gillies G.E., Pienaar I.S., Vohra S., Qamhawi Z. (2014). Sex differences in parkinson’s disease. Front. Neuroendocrinol..

[39] Rodriguez-Perez A.I., Borrajo A., Valenzuela R., Lanciego J.L., Labandeira-Garcia J.L. (2015). Critical period for dopaminergic neuroprotection by hormonal replacement in menopausal rats. Neurobiol. Aging.

[40] Anderson L.I., Leipheimer R.E., Dluzen D.E. (2005). Effects of neonatal and prepubertal hormonal manipulations upon estrogen neuroprotection of the nigrostriatal dopaminergic system within female and male mice. Neuroscience.

[41] Callier S., Le Saux M., Lhiaubet A.M., Paolo T.D., Rostène W., Pelaprat D. (2002). Evaluation of the protective effect of oestradiol against toxicity induced by 6-hydroxydopamine and 1-methyl-4-phenylpyridinium ion (mpp+) towards dopaminergic mesencephalic neurones in primary culture. J. Neurochem..

[42] Karakaya S., Kipp M., Beyer C. (2007). Oestrogen regulates the expression and function of dopamine transporters in astrocytes of the nigrostriatal system. J. Neuroendocrinol..

[43] Simpkins J.W., Yang S.-H., Sarkar S.N., Pearce V. (2008). Estrogen actions on mitochondria—physiological and pathological implications. Mol. Cell. Endocrinol..

[44] Rizzuto R., De Stefani D., Raffaello A., Mammucari C. (2012). Mitochondria as sensors and regulators of calcium signalling. Nat. Rev. Mol. Cell Biol..

[45] Quinlan C.L., Perevoshchikova I.V., Hey-Mogensen M., Orr A.L., Brand M.D. (2013). Sites of reactive oxygen species generation by mitochondria oxidizing different substrates. Redox Biol..

[46] Lejri I., Grimm A., Eckert A. (2018). Mitochondria, estrogen and female brain aging. Front. Aging Neurosci..

[47] Mo S.J., Axelsson J., Jonasson L., Larsson A., Ögren M.J., Ögren M., Varrone A., Eriksson L., Bäckström D., Af Bjerkén S. (2018). Dopamine transporter imaging with [18 f] fe-pe2i pet and [123 i] fp-cit spect—A clinical comparison. EJNMMI Res..

[48] Chen Y.H., Hsieh T.H., Kuo T.T., Kao J.H., Ma K.H., Huang E.Y.K., Chou Y.C., Olson L., Hoffer B.J. (2019). Release parameters during progressive degeneration of dopamine neurons in a mouse model reveal earlier impairment of spontaneous than forced behaviors. J. Neurochem..

[49] Chen Y.-H., Harvey B.K., Hoffman A.F., Wang Y., Chiang Y.-H., Lupica C.R. (2008). MPTP-induced deficits in striatal synaptic plasticity are prevented by glial cell line-derived neurotrophic factor expressed via an adeno-associated viral vector. FASEB J..

[50] Good C.H., Wang H., Chen Y.-H., Mejias-Aponte C.A., Hoffman A.F., Lupica C.R. (2013). Dopamine d4 receptor excitation of lateral habenula neurons via multiple cellular mechanisms. J. Neurosci..

[51] Chen Y.-H., Huang E.Y.-K., Kuo T.-T., Hoffer B.J., Miller J., Chou Y.-C., Chiang Y.-H. (2017). Dopamine release in the nucleus accumbens is altered following traumatic brain injury. Neuroscience.

[52] Cho H.-Y., Reddy S.P., Kleeberger S.R. (2006). Nrf2 defends the lung from oxidative stress. Antioxid. Redox Signal..

[53] Kawagoe K.T., Zimmerman J.B., Wightman R.M. (1993). Principles of voltammetry and microelectrode surface states. J. Neurosci. Methods.

[54] Wightman R.M., Zimmerman J.B. (1990). Control of dopamine extracellular concentration in rat striatum by impulse flow and uptake. Brain Res. Rev..

[55] Sabeti J., Gerhardt G.A., Zahniser N.R. (2002). Acute cocaine differentially alters accumbens and striatal dopamine clearance in low and high cocaine locomotor responders: Behavioral and electrochemical recordings in freely moving rats. J. Pharmacol. Exp. Ther..

